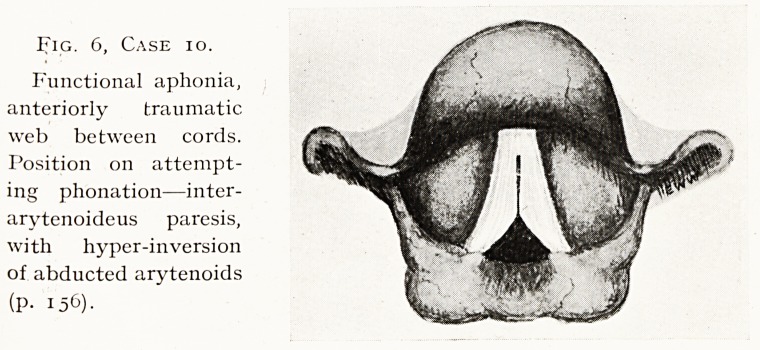# Hoarseness: The Importance of Early Laryngoscopy

**Published:** 1923-07

**Authors:** E. Watson-Williams

**Affiliations:** Registrar Ear, Nose and Throat Department, Bristol Royal Infirmary; Aural Surgeon, Ministry of Pensions Hospital, Bath


### HOARSENESS: THE IMPORTANCE OF EARLY LARYNGOSCOPY.

BY

E. WATSON-WILLIAMS, M.C., B.A., M.B. (Cantab.), Ch.M. (Bristol), F.R.C.S.E.

*Registrar Ear, Nose and Throat Department, Bristol Royal Infirmary; Aural Surgeon, Ministry of Pensions Hospital, Bath.*

---

HOARSENESS which lasts beyond quite a few days demands an examination of the larynx; clearly this part is affected whatever the condition elsewhere. When transitory this symptom is usually of slight importance, but when persistent it cannot be lightly regarded. Especially is this so when hoarseness is the sole trouble. "Hoarseness alone, without pain or any other symptom, may be the only portent of an early tuberculosis, laryngeal cancer, tabes dorsalis, aortic aneurysm or other affection, foreshadowing the urgent need of vigorous therapeutic measures." [1]

CASE I.—**Aneurysm.** G. H. H., male, æt. 50, clerk. Came to me in October, 1922, complaining of hoarseness of three months duration, without any other symptoms; onset was sudden, not associated with any disturbance of general health.

*Examination.*—Paralysis of left vocal cord, nothing else abnormal. Careful clinical examination failed to elicit anything to explain this, but a skiagram showed aneurysm of the aortic arch.

The examination should be early, quite at the beginning of the trouble. As soon as it becomes clear that the hoarseness is not rapidly clearing up we must seek if possible an explanation, before progress of disease has produced other signs indicative, perhaps, of irreparable damage. Indirect laryngoscopy is painless and simple; even children, if docile, can be examined without trouble—my youngest patient this year was only four years old. Yet this essential inspection is sometimes quite unaccountably deferred.

CASE 2.—**Trauma of Larynx.** A. P. R., male, æt. about 30. Complains of hoarseness since being wounded in throat four years earlier. The bullet had been removed by operation. Voice only hoarse whisper with periods of complete aphonia.

*Examination.*—Left vocal cord entirely missing. Left arytenoid fixed. Right side almost normal. This patient said he had been having treatment by suggestion, etc., for over two years for functional aphonia. Further treatment not advised.

Admitted that this instance is exceptional, yet it seems not very uncommon for examination to be delayed for a considerable period.

The eight remaining cases are examples from those which came under my care during the first four months of this year. In all hoarseness had been present for quite a long time before the larynx was examined; in several this was only done because further symptoms appeared. The notes of each case indicate for what reason an early examination was important.

CASE 3.—**Granuloma of Larynx.** Fig. 1. F. B., male, æt. 35, labourer. Complains of hoarseness four years, following exposure to mustard gas. No pain, recently periods of aphonia, increasing dyspnœa on exertion. Hoarseness severe and increasing.

*Examination.*—A greyish-pink, smooth, faintly lobulated mass, attached to anterior subglottic region, coming between cords on expiration. Diagnosed "fibroma." I removed the tumour by endoscopic operation. It measured about half an inch each way, and on section proved to be a granuloma. This patient had been for over a year having treatment for "D. A. H." He was shown to the Medico-Chirurgical Society before operation (February, 1923) and after, when the voice was good and the man very satisfied (April).

CASE 4.—**Hypertrophic Laryngitis.** Fig. 2. P. P., male, æt. 28, miner. Complains of hoarseness four years, getting worse; attributed to exposure; no other trouble except dysentery in the past.

Fig. 1, Case 3.

Granuloma of larynx. A greyish-pink faintly obulated mass, attached to anterior subglottic region (p. 154). Deep inspiration.

[All the illustrations represent appearances on *indirect* laryngoscopy.]

Fig. 2, Case 4.

Hypertrophic Laryngitis. On left vocal cord two prominent white horns. On right cord and posteriorly also on left, infiltrated areas. Elsewhere scars of old ulceration, some recent ulceration. Posteriorly some subglottic œdema (p. 154). Deep inspiration.

Fig. 3, Case 7.

Carcinoma of right vocal cord, almost entirely hiding the cord. Right arytenoid and false cord are fixed. The growth was found to extend into aryepiglottic fold (p. 155). Deep inspiration.

Fig. 4, Case 8.

Tuberculosis of larynx. Ulceration of left false cord and arytenoid region and of left vocal cord. Injection of right vocal cord. Inflammation and tubercle formation (very large) of epiglottis. Œdema of arytenoid and cricoid regions. Quiet respiration (p. 155).

Fig. 5, Case 9.

Papilloma of larynx, attached anteriorly, mainly subglottic but involving both vocal cords. An "infantile" epiglottis. Deep inspiration (p. 156).

Fig. 6, Case 10.

Functional aphonia, anteriorly traumatic web between cords. Position on attempting phonation—inter-arytenoideus paresis, with hyper-inversion of abducted arytenoids (p. 156).

*Examination.*—A most extraordinary larynx. Two large white horns on left vocal cord impinged on phonation on a dull red semi-pedunculated mass of granulations on the right. Farther back old and recent infiltration and ulceration caused extensive deformity of the cords. Wassermann negative. This man had received no benefit from a course of several months of inhalations, etc. Operation declined.

CASE 5.—**Syphilis of Larynx.** R. O'M., male, æt. 48, seaman. Complains of hoarseness for " a good time." During last six weeks severe, with pain on speaking and on swallowing. Pale, thin, weak, ill.

*Examination.*—Both vocal cords red, swollen and ulcerated. General œdema of arytenoid regions and cricoid. Wassermann strongly positive.

There were no other signs ; a completely negative history. On galyl and iodide the larynx healed completely, but with some permanent damage.

CASE 6.—**Laryngeal Tuberculosis.** W. C., male, æt. 24, quarryman. Complains of hoarseness nine months, getting worse. Pain on swallowing recently, none on speaking. Cough and sweating at night. Wasting.

*Examination.*—General congestion of larynx. Deep ulceration of right false cord and left aryepiglottic fold. Right vocal cord slightly ulcerated posteriorly, left shows infiltration of corresponding region. Diagnosis : tuberculous laryngitis ; Wassermann negative ; sputum swarming with tubercle bacilli ; old and active tuberculosis of both lungs. Prognosis very bad. This man had been having medicine for sore throat for five months, and was living with wife and two small children.

CASE 7.—**Carcinoma of Larynx.** Fig. 3. W. H., male, æt. 50, labourer. Complains of hoarseness four months, recently slight pain on speaking ; a little hoarseness for several months previously.

*Examination.*—Right vocal cord almost hidden by a reddish nodular growth with slight tendency to ulceration. Right arytenoid fixed, false cord rather full. No glands. Pathological report, " Squamous carcinoma." At the operation I had to remove all the right half of larynx within the ala of the thyroid cartilage, including the arytenoid. So far he has done well (third month). If this man had come under treatment earlier the operation would have been simpler, the risks less, and the prognosis as regards recurrence better.

CASE 8.—**Laryngeal Tuberculosis.** Fig. 4. S. L., female, æt. 24. Complains of hoarseness with cough ten weeks.

Sore throat six weeks. Recently pain on speaking or swallowing, much expectoration. She is weak, thin, wasting.

*Examination.*—Deep ulcers of left aryepiglottic fold and false cord ; left vocal cord bright red, raw ; epiglottis swollen, crimson, showing two large tubercles near margin ; mucosa generally pale and œdematous. Diagnosis, tuberculosis of larynx, sputum negative. Marked signs in right pulmonary apex and elsewhere. This woman had been having medicine for cough for ten weeks. In 1922 she had been laid up with pleurisy. She was looking after husband and three small children, was pregnant, and was literally dying of consumption.

CASE 9.—**Papilloma of Larynx.** Fig. 5. P. S., female, æt. 16. Complains of hoarseness for six years, getting steadily worse, recently some dyspnœa. When three years old she had had an operation on throat. No pain.

*Examination.*—An extensive papilloma springing from anterior part of larynx, mainly subglottic but involving both cords and coming between them on attempting phonation ; more than half the glottic aperture is obstructed.

The bulk of the growth I removed by endoscopic operation ; the remainder is being dealt with ; the larynx has probably suffered irreparable damage as the result of the parents' neglect.

CASE 10.—**Functional Aphonia.** Fig. 6. H. J. S., male, æt. 40, labourer. Complains of hoarseness since an operation for removal of bullet in larynx in 1918. Told then that he would never recover voice ; has hoarse whisper.

*Examination.*—Larynx practically normal ; a web unites anterior ends of vocal cords, but does not interfere with perfect movements. On attempting phonation the arytenoids are adducted (normally) then abducted and hyperinverted ; interarytenoideus paresis, a stigma of functional trouble.[2] Under ether he phonated.

Diagnosis : functional condition with organic basis. Treatment declined. Prognosis at this late stage doubtful.

I am indebted to Mr. J. P. I. Harty, F.R.C.S., for permission to use the notes of Cases Nos. 6, 7, 8 and 9.

REFERENCES.

[1] P. Watson-Williams, "Clinical Import of Hoarseness," *Practitioner*, 922, cviii. 154.

[2] P. Watson-Williams, "Diseases of Upper Respiratory Tract," 1894, p. 167.

## Figures and Tables

**Fig. 1, Case 3. f1:**
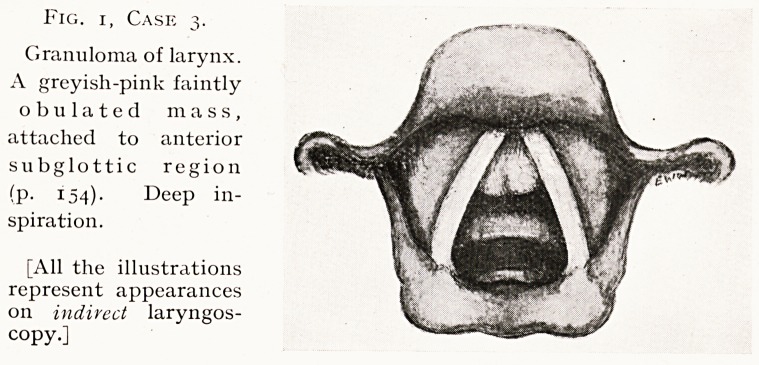


**Fig. 2, Case 4. f2:**
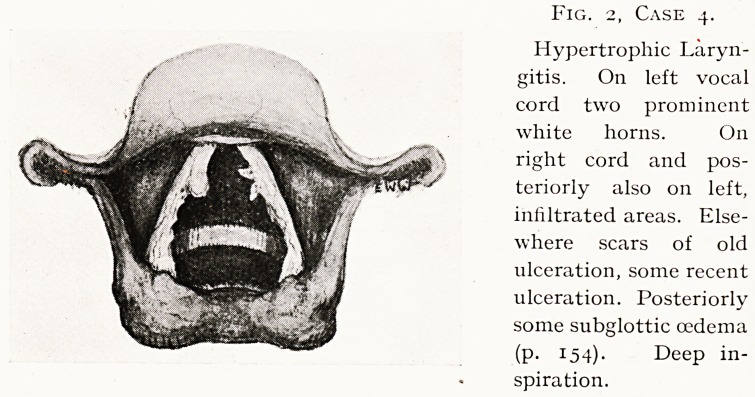


**Fig. 3, Case 7. f3:**
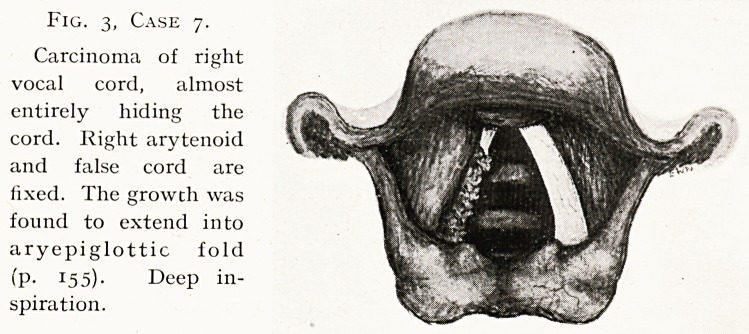


**Fig. 4, Case 8. f4:**
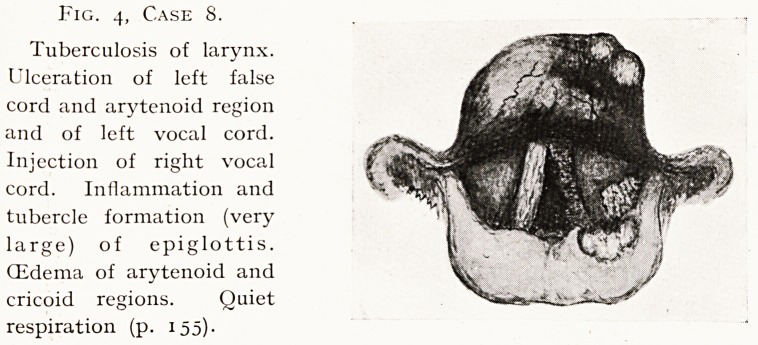


**Fig. 5, Case 9. f5:**
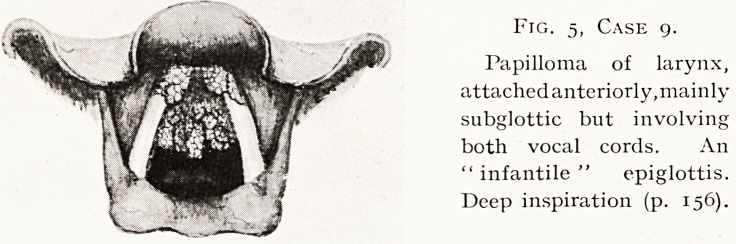


**Fig. 6, Case 10. f6:**